# Solution-Based Synthesis and Characterization of Cu_2_ZnSnS_4_ (CZTS) Thin Films

**DOI:** 10.3390/molecules24193454

**Published:** 2019-09-23

**Authors:** Ubaidah Syafiq, Narges Ataollahi, Rosa Di Maggio, Paolo Scardi

**Affiliations:** 1 Department of Civil, Environmental and Mechanical Engineering, University of Trento, Via Mesiano, 77, 38123 Trento, Italy; muhammad.mustaffa@unitn.it (U.S.); narges.ataollahi@unitn.it (N.A.); rosa.dimaggio@unitn.it (R.D.M.); 2 Solar Energy Research Institute, National University of Malaysia (SERI-UKM), Bangi 43600, Selangor, Malaysia

**Keywords:** Cu_2_ZnSnS_4_, thin film, spin coating, morphology, optical properties, phase change, electrical properties

## Abstract

Cu_2_ZnSnS_4_ (CZTS) ink was synthesized from metal chloride precursors, sulfur, and oleylamine (OLA), as a ligand by a simple and low-cost hot-injection method. Thin films of CZTS were then prepared by spin coating, followed by thermal annealing. The effects of the fabrication parameters, such as ink concentration, spinning rate, and thermal treatment temperatures on the morphology and structural, optical, and electrical properties of the films were investigated. As expected, very thin films, for which the level of transmittance and band-gap values increase, can be obtained either by reducing the concentration of the inks or by increasing the rate of spinning. Moreover, the thermal treatment affects the phase formation and crystallinity of the film, as well as the electrical conductivity, which decreases at a higher temperature.

## 1. Introduction

Kesterite, or CZTS, is a quaternary sulfide containing copper (Cu), zinc (Zn), tin (Sn), and sulfur (S) with the formula (I)_2_(II)(III)(IV)_4_. As a semiconducting compound, kesterite has p-type conductivity [1] and can withstand temperature up to 800 °C [2]. It was initially considered as an adsorbing material in thin-film solar cells to replace Cu(In,Ga)Se_2_ (CIGS), whose elements are expensive and scarcely available. Indeed, CZTS exhibits good light-harvesting performance, with an optimal band gap energy around 1.5–1.6 eV [3,4], given by conduction and valence bands around −3.8 and −5.2 eV, respectively [5,6]. The external quantum efficiency (EQE) for CZTS solar cells shows that the light-harvesting process starts from about 400 nm up to more than 800 nm [7] radiation wavelength.

Nowadays, fabrication of films from CZTS suspension through the ink-based approach has attracted considerable research interest as a simple, economic, and industrially scalable route, compared to the more expensive vacuum deposition methods. This method allows better material utilization with high throughput. Moreover, an ink-based approach lets simple deposition methods, such as spin coating, dip coating, spray coating, screen printing and ink-jet printing, be used [8,9,10]. Inks were prepared by solvo-thermal methodology, one of the simplest and fastest ways to synthesize high-quality CZTS nanoparticles on the gram scale, which involves the injection of a sulfur solution into a hot mixture of metal precursors dissolved in a solvent with a high boiling point [11]. In this regard, and despite the progresses in the production process, a fine control of the composition, shape, and size of CZTS nanoparticles requires complex metal sources, solvents, and ligands. Several studies demonstrated that solvents can affect the final shape of CZTS nanoparticles [12,13], whereas the sulfur source and reaction temperature can affect the crystal structure [14]. Actually, a simple approach based on metal chlorides, sulfur powder and oleylamine (OLA), playing a double role of solvent and complexing agent, has been already proposed [4,15] to produce quaternary CZTS nanoparticles on the gram scale. A similar method has been successfully employed for producing CZTS nanoparticles for solar cell applications, with a device efficiency up to 10.3 % [16].

Where the electrical properties of binary and ternary semiconductors have been studied for years [17,18,19], those of CZTS have been investigated more recently [20,21], especially taking into account their temperature dependence. In general, the electrical conductivity of CZTS films is due to Mott variable-range hopping and nearest neighbor hopping mechanisms below −125 °C. Above that temperature, the conductivity increases because of either thermionic emission of carriers over grain boundary barriers or the release of carriers from defect states [22]. In summary, two distinct modes of electronic transport are observed: hopping conduction (less than −125 °C) and thermally activated conductivity (greater than −125 °C). For ink-based CZTS thin films, it is reported that an increase in thermal treatment (TT) temperature increases the conductivity of the film, but evidence on this important aspect of the production process is still limited [21].

So far, properties of micrometric and sub-micrometric (>0.5 μm) CZTS thin films fabricated using several different methods have been extensively studied [7,23,24,25,26] with the purpose of being the absorber layer in solar cell. There are also studies on CZTS thin films with <0.5 μm thickness, in which they are used as a transport layer in a perovskite solar cell [27,28,29]. However, their focus is on the application of the thin film in the solar cell, with limited information on properties. Thus, this study focuses on the influence of deposition parameters and thermal treatment on the morphology and structural, optical, and electrical properties of CZTS thin film synthesized using a hot-injection method, which has not, to the best of our knowledge, been reported in the literature. The main novelty of this study is the effect of thermal treatment on the crystal structure, which further affects other properties of the films at <0.5 μm thickness. The results of this study can provide some insights on the versatility of CZTS, not just as a traditional absorber layer, but as transport layer.

## 2. Results and Discussion

### 2.1. CZTS Ink Concentration and Spin Rate 

**Figure 1:**
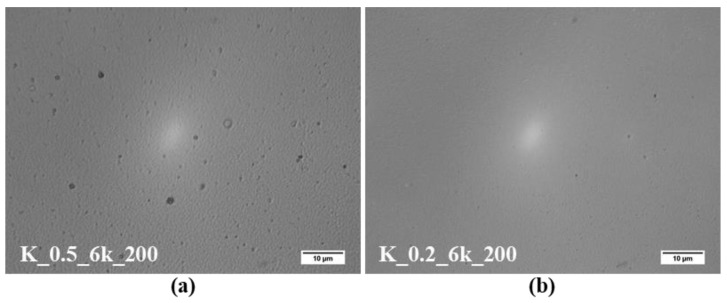

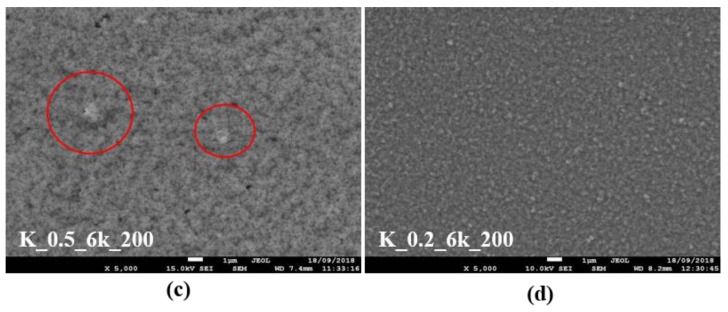
Surface of the Cu_2_ZnSnS_4_ (CZTS) thin film: (**a**,**b**) optical microscopy and (**c**,**d**) scanning electron microscopy (SEM) prepared by using ink with concentrations of nanoparticles at 0.5 and 0.2 g/mL, respectively.

The first parameter studied was ink concentration. Figure 1a,b shows the images from optical microscopy of the CZTS thin film with 0.5 and 0.2 g/mL of CZTS nanoparticles in toluene, respectively; the lower the concentration, the lower the number of surface irregularities. The increase of CZTS nanoparticles per unit volume increased the possibility of agglomeration, which reduced the surface energy [30,31]. On the contrary, CZTS nanoparticles were better dispersed in a more diluted solution, thus providing a smooth and continuous layer of CZTS.

Figure 1c,d shows the corresponding SEM pictures of the surface morphology of CZTS thin films with different ink concentrations deposited on fluorinated tin oxide (FTO) glass. In general, the obtained thin films consisted of continuous layers of CZTS nanoparticles that homogenously covered the entire surface of FTO glass. However, some agglomeration, highlighted by red circles, was visible on CZTS thin films prepared with 0.5 g/mL ink. After deposition, the higher packing density forced some nanoparticles to form agglomerates, resulting in a lower surface energy [32].

**Figure 2:**
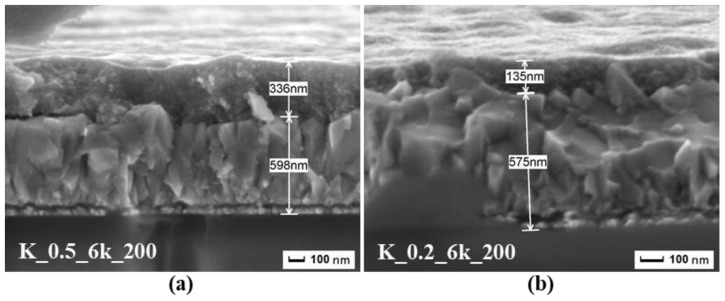
Cross-section of the CZTS thin film on fluorinated tin oxide (FTO) glass by SEM, prepared with ink having concentrations of (**a**) 0.5 g/mL and (**b**) 0.2 g/mL, respectively.

SEM observations of the cross-section of the thin films with different ink concentrations (Figure 2) allowed us to evaluate the thickness of the thin films. The average thickness observed for CZTS thin films with 0.5 and 0.2 g/mL concentrations were ~336 and ~135 nm, respectively. As expected, the higher the ink concentration, the thicker the thin films were.

**Figure 3:**
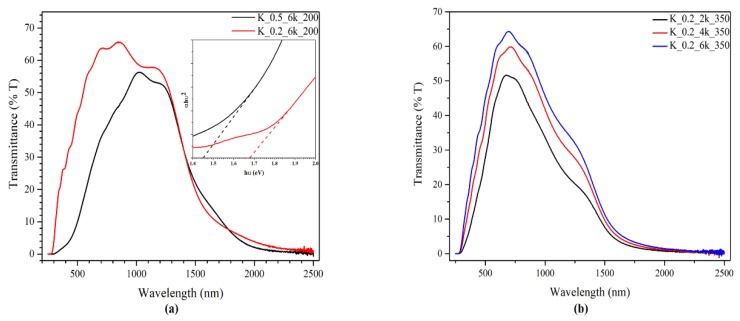
UV–vis spectroscopy of the CZTS thin film (**a**) prepared by ink having concentrations of 0.5 g/mL (red line) and 0.2 g/mL (black line), the insert is Tauc’s plot of corresponding CZTS thin film, and (**b**) at different spin rates: black line, 2000 rpm; red line, 4000 rpm; and blue line, 6000 rpm.

Figure 3a shows the ultraviolet–visible (UV–vis) spectroscopy results with different ink concentrations. The CZTS thin film with 0.2 g/mL ink has a higher transmittance level compared to 0.5 g/mL ink. UV–vis spectroscopy (Figure 3b) of the CZTS thin film deposited on the FTO glass shows that with an increasing spin rate, the transmittance correspondingly increases. According to Beer–Lambert’s law, a decrease in thickness of a material will decrease its absorption, thus causing an increase in transmittance [33,34]. This result confirms that the lower the ink concentration and the higher the spin rate are, the thinner the CZTS film will be. 

Tauc’s plot of CZTS thin films with different ink concentrations are shown in the insert of Figure 3a. The band gaps for CZTS thin films were ~1.45 and ~1.68 eV, for inks having nanoparticle concentrations of 0.5 and 0.2 g/mL, respectively. 

Although the band gaps obtained were still in accordance with the band gap range of CZTS reported in the literature [3,5,6], the decrease in band gap with an increasing concentration can be related to the greater thickness of CZTS thin film deposited. There is a possibility of structural defects in the film, which could give rise to localized states near the conduction band. In the case of thick films, these localized states may as well merge with the conduction band, resulting in a reduction of the bandgap [35]. 

### 2.2. TT Temperature

**Figure 4:**
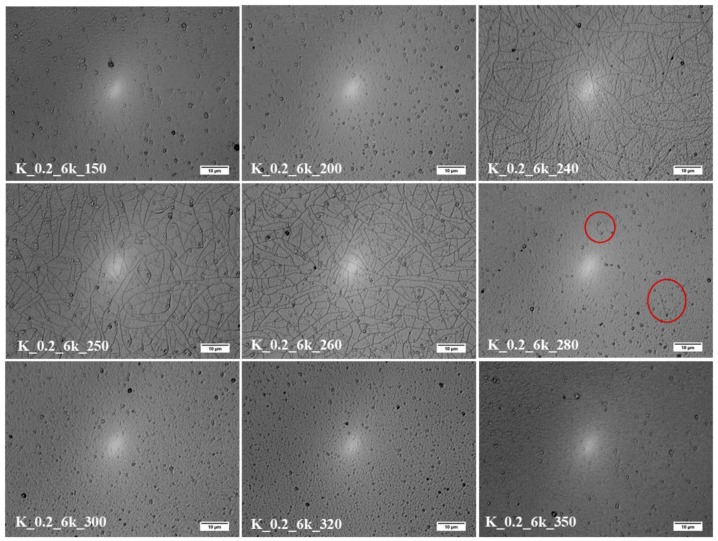
Morphology of the CZTS thin film surface by optical microscope at different TT temperatures indicated on each micrograph.

The effect of TT temperature on CZTS thin film samples, obtained from 0.2 g/mL ink spread at a 6000 rpm spin rate, was studied in terms of its surface morphology, grain growth, optical behavior, and electrical properties. Figure 4 shows the micrograph of the surface of CZTS thin films treated at temperatures from 150 to 350 °C. The treatments in the range 240–280 °C seemed to induce cracks in samples, probably because of excessive drying and the shrinkage rate [36,37]. In fact, the samples treated at 150 and 200 °C appeared smooth and continuous. Also, the thin films treated at 300, 320, and 350 °C did not show cracks because, at those temperatures, the CZTS grain growth [38] and phase changes compensated the shrinkage and avoided crack formation.

**Figure 5:**
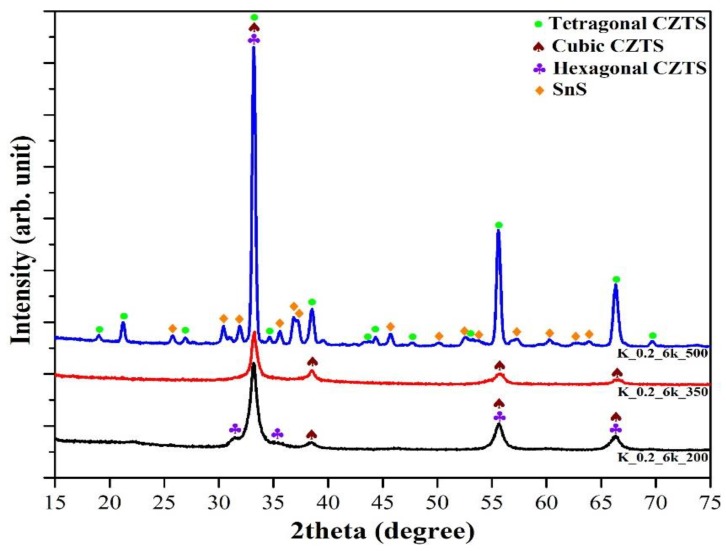
XRD spectra of CZTS thin films treated at 200 °C (black line), 350 °C (red line), and 500 °C (blue line). Identified phases are indicated by corresponding symbols.

In Figure 5, the X-ray diffraction (XRD) spectra of the samples treated at 200, 350, and 500 °C are shown, demonstrating that different CZTS phases formed. At TT 200 °C, a mix of cubic (distinctive peaks at 2θ = ~33.2°, ~38.5°, ~55.7°, and ~66.3°) and hexagonal, also known as wurtzite (at 2θ = ~31.4°, ~35.3°), CZTS phases formed [39]. At TT 350 °C, only cubic CZTS phase formed [40]. At TT 500 °C, a tetragonal (distinctive peaks at 2θ = ~18.9°, ~21.3°, and ~38.5°) [15,41] peak formed, and also a secondary phase, tin (II) sulfide (SnS), formed having main peaks at 2θ = ~25.8°, ~30.4°, ~31.9°, ~35.6°, ~36.8°, ~37.2°, and ~45.7°. It was demonstrated [40] that when the nanocrystals of CZTS are very small, they can be cubic or hexagonal, and with an increasing size, one observes cubic only, which evolve to the thermodynamically stable tetragonal form when crystals are big enough. 

The crystallization process, and especially the grain growth, as demonstrated by the sharpness of peaks, was strongly promoted at 500 °C, while the formation of a secondary phase (SnS) might be due to unbalanced stoichiometry [42]. 

**Figure 6:**
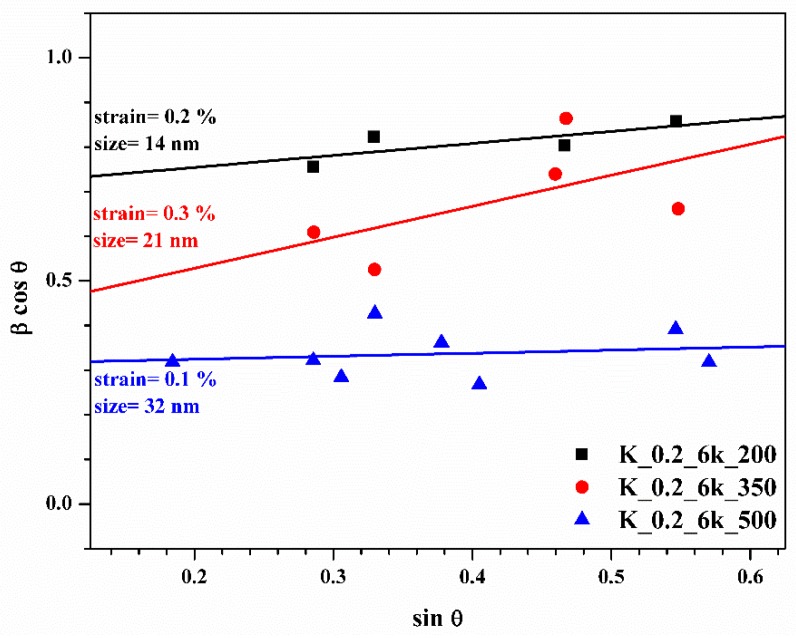
The Williamson–Hall (W-H) analysis (β, integral breadth of diffraction peaks; θ, Bragg diffraction angle) of CZTS thin films treated at 200 °C (black line), 350 °C (red line), and 500 °C (blue line).

The Williamson–Hall (W-H) analysis [43,44] was performed on XRD data to study the effect of the TT temperature on the size of the nanocrystals and on the microstrain. The latter was due to the surface of the nanocrystals but can also be caused by stoichiometric fluctuations [45]. As shown in Figure 6, the microstrains at TT 200 °C and TT 350 °C are greater than that at TT 500 °C. The reduction of the microstrain is probably related to the consequent increase in the size of the crystals, since OLA, which acts as ligand, is decomposed at TT 500 °C, resulting in further grain growth.

**Figure 7:**
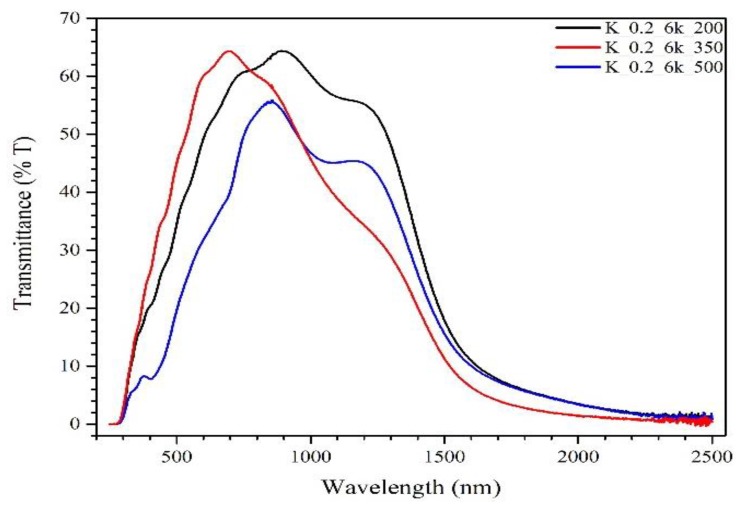
UV–vis spectroscopy of CZTS thin films at different TT temperatures: black line, 200 °C; red line, 350 °C; and blue line, 500 °C.

Figure 7 shows the UV–vis spectroscopy of CZTS thin films deposited on FTO glass under three different TTs. The shift of the transmittance curve towards the visible range for the film treated at 350 °C compared to 200 °C was due to the absence of a wurtzite phase. As reported [46], films containing wurtzite phase show higher absorptions in the visible range. Furthermore, the transmittance of the film treated at 500 °C showed a shift toward higher wavelengths due to Sn loss [47]. The maximum peak of transmittance for TT 200 °C and TT 350 °C was ~65%; however, it was reduced to ~55% for TT 500 °C. This was due to grain growth, which leads to an increase in surface roughness, causing increased scattering and absorption of light [48].

From the UV–vis spectroscopy data, the band gaps of the thin films were determined to be at ~1.62 eV for TT 200 °C, ~1.57 eV for TT 350 °C, and ~1.73 eV for TT 500 °C, respectively, which is in good agreement with those reported in the CZTS literature [3,5,6]. However, the slight differences observed among the samples might be due to the different phases present in each sample. In fact, samples at TT 200 °C and TT 350 °C showed a similar band gap because a cubic CZTS phase was prevalent in both, although the wurtzite in the TT 200 °C sample favored a shift of the band gap towards a higher value [49]. The presence of multiple and spurious phases favors the increase of the optical absorption edge towards higher energy values [50] and is confirmed by the high band gap value of the sample treated at 500 °C.

Electrical properties of CZTS thin films were studied at different TT temperatures (TT 200 °C, 350 °C, and 500 °C) by Hall effect measurements. Results shown in Table 1 refer to CZTS thin films deposited on soda-lime glass (SLG) as FTO interfered with the measurement of such thin layers. The value of resistivity decreases significantly as the TT temperature increases to 500 °C, which is in agreement with literature [51,52]. Phase changes and microstructures are known to play a role in the electrical properties of CZTS. Resistivity of tetragonal CZTS is lower than other CZTS phases [53], and grain growth and refining are promoted by high-temperature TTs, reducing the amount of defects [21]. However, the large difference observed in resistivity and carrier concentration most probably depends on the presence of organic residuals in the lower temperature TTs [15]. 

While resistivity and carrier concentration obtained at 500 °C were better than literature values [54,55], mobility was lower. It has been already observed that carrier mobility is strongly dependent on the microstructure and secondary phase [52], like SnS that formed at 500 °C. Since the carrier mobility is inversely proportional to the resistivity, eliminating the SnS secondary phase will increase carrier mobility, further reducing the resistivity and, thus, improving the overall electrical properties of the CZTS thin film.

**Figure 8:**
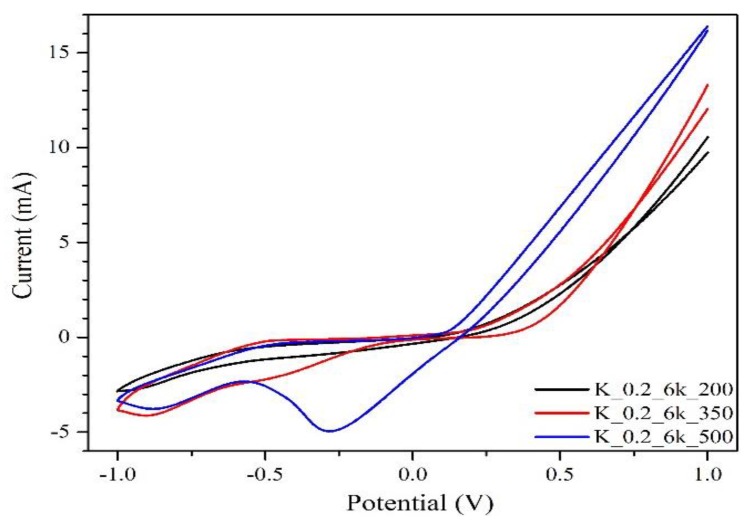
CV of CZTS thin films with different TT temperatures: black line, 200 °C; red line, 350 °C; and blue line, 500 °C.

The better electrical properties of the film made at 500 °C was also demonstrated by cyclic voltammetry (CV) measurements. The cathodic current density (J_pc_) was determined by the difference between the maximum peak current density and the baseline of charging current density [56]. It is worth noting that the peak at the negative potential corresponds to the reaction I_3_^−^ + 2e^−^ → 3I^−^, where the current density can be related to the rate of reduction of I_3_^–^ to I^–^, occurring at the CZTS electrode. As shown in Figure 8, TT 500 °C had the highest current density, which can be explained by a better electrocatalytic ability of the electrode [57]. In general, CV confirmed the Hall effect results, demonstrating the importance of high-temperature TTs to make CZTS thin films with better electrical properties. The question that still remains is how to avoid formation of secondary phases, which are detrimental to the mobility of the carriers. 

## 3. Materials and Methods 

Copper (II) chloride dihydrate (CuCl_2_·2H_2_O, >99.0%), zinc chloride (ZnCl_2_, >98.0%), and tin (II) chloride (SnCl_2_, 98%) were purchased by Sigma-Aldrich Inc. and dehydrated in vacuum at 200 °C. Sulfur (S), oleylamine (OLA, 70%), toluene (99.9%), and ethanol (>99%) were also provided by Sigma-Aldrich Inc. and used without further purification.

### 3.1. Synthesis of CZTS Nanoparticles Ink

CZTS nanoparticles ink was prepared according to the method described by Ataollahi et al. [15]: 0.538 g of CuCl_2_·2H_2_O, 0.414 g of ZnCl_2_, and 0.410 g of SnCl_2_ were mixed into a 100 mL three-neck round-bottom flask containing 6.6 mL of OLA. The system was connected to a Schlenk line apparatus to carry out the whole process in standard, air-free conditions. OLA was added to work both as a solvent and as a capping agent for the nanoparticles. The mixture was heated first to 130 °C, and at this temperature the flask was degassed and refilled with nitrogen several times and then kept under vacuum. About 8 mL of a sulfur/OLA 1 M solution (prepared by dissolving 0.449 g S in 10 mL OLA) was rapidly injected in the hot solution at 270 °C under fast stirring and nitrogen (N_2_) flux. The mixture was kept at 270 °C for 30 min after the injection and then cooled slowly to room temperature. The obtained suspension of nanoparticles was washed with a mixture of 5 mL toluene and 25 mL ethanol and centrifuged for 10 min at 4000 rpm to separate the solvent from the nanoparticles. Finally, the CZTS nanoparticles were extracted, dispersed in toluene, and sonicated to obtain an ink with the desired concentration.

### 3.2. Deposition of CZTS Thin Film

The CZTS nanoparticles ink was deposited on SLG substrates by spin coating. Before deposition, the substrates were cleaned with soap, treated with concentrated potassium hydroxide (KOH) in ethanol, rinsed with distilled water and ethanol, then dried in N_2_ stream. The cleaning process of the FTO glass was similar, except for the treatment with KOH, where 20 μL of ink was dropped on the substrate. After spin coating, the residual toluene was removed at 150 °C for 15 min. The TT below 350 °C was carried out on a hot-plate under N_2_ atmosphere for 15 min. High-temperature TT (500 °C) was performed in a tubular furnace at a heating rate of 3 °C/min for 1 h under N_2_ atmosphere. Samples were labelled according to ink concentration (0.2 and 0.5 g/mL), spin rate (2000–6000 rpm), and TT temperature (150–500°C). 

### 3.3. CZTS Thin Film Characterization

The morphology of CZTS thin film samples was observed either by optical microscopy or by scanning electron microscopy (SEM). Optical microscopy was made on a REMET HX-1000 (Remet, Bologna, Italy) at 400× magnification, whereas for SEM we used a JEOL JSM-7001F FEG-SEM (JEOL, Tokyo, Japan) equipped with an energy-dispersive X-ray spectroscopy detector (EDXS, Oxford INCA PentaFETx3, Oxford, United Kingdom). Observations were made at 10.00–15.00 keV electron beam energy with a working distance between 5–10 mm. Surface morphology images were acquired in top-down modes, whereas a cross-sectional analysis was performed by putting the cross-section of the film on a 90° stub.

UV–vis spectroscopy of CZTS thin films was made using a Perkin-Elmer LAMBDA 950 spectrophotometer (Perkin-Elmer, Milano, Italy) equipped with a 150 mm integrating sphere. By measuring normal incidence transmittance and reflectance, the absorption coefficient was calculated using the equation

(1) $$\alpha =-\frac{1}{d}ln\left(\frac{T}{1-R}\right)$$

where:

α = absorption coefficient,
*d* = film thickness,
= normal incidence transmittance, and
= normal incidence reflectance.

From UV–vis spectroscopy data, the bandgap energy was also obtained by a linear fit of a Tauc plot.

Structural information on CZTS thin films were obtained by X-ray diffraction (XRD), using a Panalytical X’Pert MRD instrument equipped with CoKα sealed tube operated at 40 kV and 40 mA. Phase identification was supported by JADE Software (Materials Data, Inc., Livermore, CA, USA).

Hall effect measurements were made using an electrical testing platform (RH 2030 – PhysTech, Germany) with a current source range of 10^−10^–10^−2^ A, with maximum resolution of 2.5 × 10^−12^ A. The maximum output voltage was ± 20 V with the output resistance using a typical 10^13^ Ω resistor. Besides that, the voltage measurement range was 10^−6^–10^1^ V, with maximum resolution of 3 × 10^−7^ V. The instrument had a sensitivity of <1 mV and an input resistance of >10^15^ Ω. The magnetic field was provided by a standard magnet with magnetic field (B) range of 0.05–0.8 T and an achievable maximum resolution of 0.001 T. The pole diameter and gap were 45 and 0–75 mm, respectively. The resistivity value was calculated by using the formula

(2) $$\rho =\frac{1}{\sigma }=\frac{1}{q\mu n}$$

where:

*ρ* = sample resistivity (Ω∙cm),
σ = sample conductivity (Ω^−1^∙cm^−1^),
= charge constant (1.6 × 10^−19^ C),
μ = carrier mobility (cm^2^/Vs), and
= carrier concentration (cm^−3^).

Cyclic voltammetry (CV) was conducted on Princeton Applied Research (PAR) model no. 4000 A with a three-electrodes system. Silver/silver chloride (Ag/AgCl) was the reference electrode, platinum (Pt) was the counter electrode, and CZTS thin film samples were deposited on the FTO glass as working electrodes with iodide/triiodide ion (I^−^/I_3_^−^) electrolytes using a methanol solution containing 0.5 M potassium iodide (KI) and 0.5 mM I_2_. Scanning was done at a rate of 50 mV s^−1^.

## 4. Conclusions

Ink containing CZTS nanoparticles was successfully synthesized by the hot-injection method using metal chloride precursors, sulfur, and OLA. It was found that the ink concentration affected the thickness and surface morphology of the deposited film. The spin rate can be manipulated to obtain films with different thicknesses. Both a lower ink concentration and a higher spin rate produced CZTS thin films with lower thickness, higher transmittance, and better morphology. Moreover, the different thermal treatment temperatures (from 150 to 500 °C) affect more than other parameters on the overall quality, surface morphology, crystal structure, transmittance behavior, band gap energy, and electrical properties of the CZTS thin film. The increase in TT temperature induces significant phase changes (wurtzite CZTS → cubic CZTS → tetragonal CZTS) in addition to an improvement in the crystallinity degree and electrical properties. While high-temperature TTs are required to get rid of organic residuals and increase conductivity and carrier concentration, they can also lead to formation of secondary phases like SnS, which is detrimental to the mobility of the carriers. A correct balance between different requirements in thin film production still has yet to be found. The method is confirmed to be very versatile for preparing CZTS sub-micrometric thin films (< 0.5 µm) and semiconductors suitable for a transport layer in many applications such as in solar cells, diodes, and thermoelectric devices.

## Figures and Tables

**Table 1:** Hall effect measurement results of CZTS thin films with different TT temperatures.

Sample	Thickness (nm)	Resistivity, ρ (Ω·cm)	Carrier Concentration, n (cm^−3^)	Carrier Mobility, μ (cm^2^/Vs)
**TT 200 °C**	~135	4.0 × 10^4^	2 × 10^13^	13
**TT 350 °C**	~100	4.6 × 10^4^	5 × 10^12^	27
**TT 500 °C**	~90	4.1 × 10^−1^	1 × 10^21^	0.5
